# 2370. Characterizing Predictors of COVID-19 Vaccine Refusal in an Urban Southern California Jail

**DOI:** 10.1093/ofid/ofad500.1991

**Published:** 2023-11-27

**Authors:** Nazia Qureshi, Loren G Miller, Stephen Judge, Ngoc Dung Tran, Sean O Henderson

**Affiliations:** Los Angeles County Department of Correctional Health Services, Los Angeles, California; David Geffen School of Medicine at UCLA, Torrance, California; Harbor-UCLA Medical Center, Torrance, California; Los Angeles County Department of Correctional Health Services, Los Angeles, California; Los Angeles County Department of Correctional Health Services, Los Angeles, California

## Abstract

**Background:**

Correctional populations have been disproportionately affected by COVID-19, with majority of the largest single-site outbreaks being linked to jails and prisons. Vaccination is a key strategy to reduce the transmission of SARS-CoV-2 in carceral settings but can be challenging to implement due to vaccine hesitancy and medical mistrust. We sought to identify factors associated with COVID-19 vaccine refusal in the largest urban jail system in the Unites States.

**Methods:**

We retrospectively analyzed electronic health record data for individuals who were offered COVID-19 vaccination at the Los Angeles County Jail between January 19, 2021 and January 31, 2023 and used multivariable logistic regression to determine predictors of COVID-19 vaccine refusal.

**Results:**

Of the 21,424 individuals offered, 2,060 (9.6%) refused vaccination. Refusal was associated with being male ([aOR] = 2.3, 95% CI (1.9, 2.8)), age 18-34 ([aOR] = 1.2, 95% CI (1.1, 1.4), referent group: age 45-54), Black/African American race ([aOR] = 1.2, 95% CI (1.1, 1.4)), reporting ever being houseless ([aOR] = 1.2, 95% CI (1.1, 1.3)), and having a history of not receiving influenza vaccination while incarcerated ([aOR] = 2.4, 95% CI (2.0, 2.8)). When analyzing the male and female populations separately, the male-specific trends reflected those seen in the overall population, whereas the only significant predictor of vaccine refusal in the female population was not receiving influenza vaccination while in custody ([aOR] = 6.5, 95% CI (2.4, 17.6)).

**Figure:**
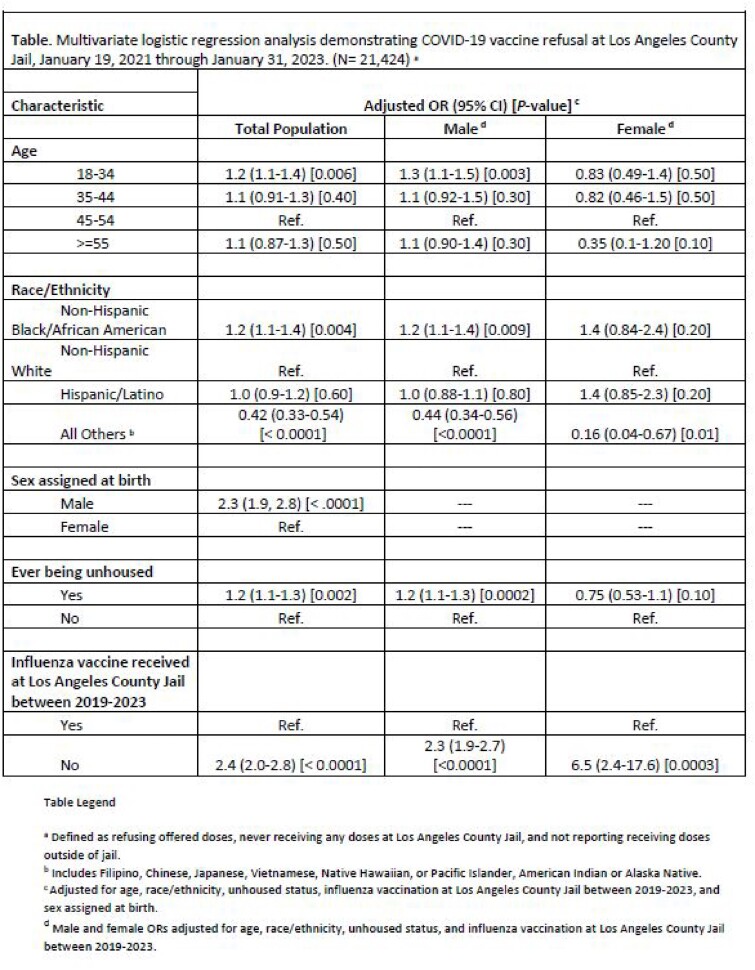

**Conclusion:**

Identifying predictors of vaccine refusal in correctional populations is an essential first step in the development and implementation of targeted interventions to mitigate vaccine hesitancy.

**Disclosures:**

**Loren G. Miller, MD MPH**, ContraFect: Grant/Research Support|GSK: Grant/Research Support|Medline: Grant/Research Support|Merck: Grant/Research Support|Paratek: Grant/Research Support

